# Oil nanoencapsulation: development, application, and incorporation into the food market

**DOI:** 10.1186/s11671-018-2829-2

**Published:** 2019-01-07

**Authors:** Camila Duarte Ferreira, Itaciara Larroza Nunes

**Affiliations:** 1 Nutrition School, Federal University of Bahia, Basílio da Gama Street, w/n, Canela. 40.110-150, Salvador, Bahia Brazil; 2 Department of Food Science and Technology, Federal University of Santa Catarina, Admar Gonzaga Highway, 1346, Itacorubi. 88034-000, Florianópolis, Santa Catarina Brazil

**Keywords:** Nanotechnology, Lipid, Nanoparticle, Oil nanoencapsulation technology, Market, Patent

## Abstract

Oils are very important substances in human nutrition. However, they are sensitive to oxygen, heat, moisture, and light. In recent years, there has been a growing interest in the modification technology of oils. Methods that modify oil characteristics and make oils suitable applications have been increasingly studied. Nanotechnology has become one of the most promising studied technologies that could revolutionize conventional food science and the food industry. Oil nanoencapsulation could be a promising alternative to increase the stability and improve the bioavailability of nanoencapsulated compounds. The occurrence of oil nanoencapsulation has been rapidly increasing, especially in the food industry. Conventional nanoencapsulation technologies applied in different oils exert a direct impact on oil nanoparticle synthesis, influencing parameters such as zeta potential, size, and the polydispersity index; these characteristics might limit the use of oils in different industries. This review summarizes oil nanoencapsulation in the food industry and highlights the technologies, advantages, and limitations of different techniques for obtaining stable oil nanocapsules; it also illustrates key opportunities for and the benefits of technological innovations and analyzes the protection of this technology through patent applications. In the last 20 years, oil nanoencapsulation has grown considerably in the food industry. Although nanoencapsulated oil products are not currently found in the food industry, there are numerous articles in the food science area reporting that oil nanoencapsulation will be a market trend. Nevertheless, different areas can apply nanoencapsulated oils, as demonstrated via patent applications.

## Introduction

Oils have an important role in human nutrition. In addition to providing calories, they act as a vehicle for fat-soluble vitamins, such as A, D, E, and K. Oils are also sources of essential fatty acids, such as linoleic, linolenic, and arachidonic acids, and they contribute to food palatability. The most expressive oil components are triglycerides, and the physical properties of these triglycerides depend on the structure and distribution of the present fatty acids [1–4].

Approximately 90% of oil production is of vegetable origin derived from the processing of seeds and is destined for human consumption. In the industry, there has been a market demand increase for oils from a wide range of natural sources, especially in food applications for the formulation of products such as cakes, biscuits, breads, margarines, and dairy products and for use in fried goods, among other applications [5, 6].

The remaining 10% of oil production is destined for animal feed production and use in several industrial processes, such as raw materials for the manufacture of fungicides, soaps, detergents, soaps, biodegradable softeners, cosmetics, and biodiesel [5].

Considering the nutritional and economic importance of edible oils, there has been a growing interest in recent years in the modification technologies of these oils. Modification technologies have been increasingly studied to alter the characteristics of the oils and make them suitable for certain applications. Researchers have found various technologies to improve food quality and safety. The involvement of nanotechnology in the food industry has led to the production of food with better thermal stability, better solubility, and novel, higher levels of oral bioavailability [7].

Nanotechnology has been suggested to positively impact the field of food science by increasing the shelf-life of food products, enabling better contaminant tracking and tracing methods, creating improved food storage strategies, and advancing the incorporation of health supplements or antibacterial agents into food. Thus, nanotechnology indeed greatly contributes to food science [7].

Nanotechnology has become one of the most promising technologies to revolutionize conventional food science and the food industry. Nanotechnology-assisted processing and packaging has proved the importance of nanotechnology in food systems. Different preparation technologies could produce nanoparticles with different physical properties; thus, these particles could be used in food [8, 9].

Encapsulation is a process in which bioactive lipid droplets are recovered by a crust or enclosed in a heterogeneous or homogeneous matrix to create small capsules [3] of nanoscales [10] with sizes less than 1000 nm, a nanometer being one billionth of one meter [11]; encapsulation has many useful properties [3]. According to Gonnet et al. [12], encapsulation is a potential approach in preserving the natural/native oil properties over time. The classic systems developed in nano or microencapsulation are based on reservoir or matricidal particles.

In addition to its benefits, nanoencapsulation is characterized by enhancing the bioavailability of the encapsulated active substance and protecting them against natural and processing effects, such as the chemical effects [13, 14], enzymatic effects, and physical instability seen during the processing of functional, nutraceutical [13], pharmaceutical, and cosmetic [3] products [10]. Encapsulation also represents a means to improve biological efficiencies, such as control of the delivery of active components and shelf-life, and could prevent the emergence of side effects [12].

Oil encapsulation may prevent or slow oxidation reactions, considering that these systems may constitute a physical-chemical barrier against prooxidant elements such as oxygen, free radicals, or ultraviolet radiation (UV) [12, 15] and widen the range of food commodities intended for enrichment purposes. Bioactive oil encapsulation, for example, represents an efficient, feasible approach in the modification of oil release, the protection from environmental oxidation reactions, an increase in physical stability, a decrease in volatility, a reduction in toxicity, an enhancement in bioactivity, and an improvement in patient compliance and convenience [3]. Specifically, in the food industry, this technology enhances processed food qualities such as flavor retention, antioxidation, shelf-life, color, and off-odor; extends food product storage time; and protects ingredients from the environment, reducing flavor loss during preservation, and controlling the release of bioactive substances [16].

Many techniques are applied for encapsulation. In general, three methods are used in the encapsulation of bioactive agents: (a) a barrier structure is created around the encapsulated agent; (b) contaminated materials are denied entry; and (c) encapsulated agents are arranged for protection against undesired detriments [17].

In many cases, nanoencapsulation begins with the production of nanoemulsions, which are systems formed by oily and aqueous phases; nanoencapsulations are emulsified through the use of, in most cases, emulsifiers. In addition, nanoemulsions are formed with small drop sizes and high surface areas [18]. Such properties grant them potential advantages over conventional emulsions, such as good physical stability and higher bioavailability [19]. Some techniques studied for obtaining oil nanoemulsion and oil nanoencapsulation include nanoprecipitation, spray drying, ionic gelation, interfacial deposition of the preformed polymer, emulsion-diffusion, emulsification-solvent evaporation, the use of liposomes, high-shear homogenization (microfluidization), spontaneous emulsification, and nanostructured lipid carriers (NLCs).

The purpose of this study is to survey the potential and current applications of oil encapsulation in the food industry, illustrating the key benefits of and opportunities for innovation and also considering future challenges, including current products in the food market and patent application. New nanoencapsulated oil products and patent applications lend promise for the use of oil in various industry sectors. Furthermore, micro- and nanoencapsulation can promote (a) a reduction in the evaporation or transfer rate of the core material to the outside environment; (b) core material protection from degradation by a reduction in the reactivity to the outside environment; (c) control of the rate of core material release, either slowly over time or at a particular time; (d) modification of the physical characteristics of the original material to allow easier handling; (e) masking of an unwanted flavor or taste of the core material; (f) separation of the mixture components that would otherwise react with one another; and (g) dilution of core materials when only small amounts are required to achieve uniform dispersion in the host material [17].

### General Nanoencapsulation of Colloidal Nanoparticles

The synthesis of nanoparticles and other nanostructures has received considerable attention in recent years since their properties, such as optical, mechanical, and chemical properties, depend strongly on their size, geometric structures, and components, which are quite different from those of bulk materials [20, 21].

Nanoparticles are colloidal particles. The two most common types of colloidal delivery systems with sufficiently small particles to achieve optical transparency are microemulsions and nanoemulsions. Both systems contain small particles (*d* < 200 nm). One of the main advantages of nanoemulsions over microemulsions is that they require considerably less surfactant to form them. Food-grade nanoemulsions can be formed by high-energy methods (such as high-pressure homogenization or sonication) or low-energy methods (such as phase inversion temperature, spontaneous emulsification, or emulsion phase inversion) [22].

Colloidal particles can be produced for different proposes, such as applications in metal [20], biomedical [23], medical [24], sensor [25], optics [25], flavoring, beverage, repellent, fragrance, and cosmetic products; used for their medicinal properties [26], food [22], and used in essential oils (EOs) for different purposes [27, 28].

Colloidal delivery systems, including emulsions, can be designed to incorporate polyunsaturated fatty acids (PUFA) into aqueous environments to improve the system’s oxidative stability. Most of these emulsion-based delivery systems contain particles that have dimensions similar to the wavelength of light, and they therefore scatter light strongly, leading to high turbidity or opacity. For certain applications, it is advantageous to use a transparent delivery system so that it can be incorporated into optically clear food or beverage products, such as some fortified waters, soft drinks, and dressings [22].

Regarding soft drinks, Ziani et al. [29] formed colloidal dispersions containing lemon oil, a nonionic surfactant (Tween 80), and a buffer (pH 2.6). This study provides useful information for the rational design of food-grade colloidal delivery systems for encapsulating flavor oils and other functional lipids in foods and beverages.

Solid lipid nanoparticles (SLNs) have gained increased attention in the pharmaceutical and food industries because of their ability to overcome the deficiencies of both microcapsules and the previously mentioned nanoscale colloidal carrier systems. SLN are the latest generation of nanoscale encapsulation systems, combining the advantages given from the parent liquid nanoemulsions or microemulsions of high dissolution velocities associated with high permeabilities of the active compound through the gut wall with the simultaneous solutions to the existing problems associated with the physical and chemical stability of the encapsulated compound and the ease of handling [30].

Lipid nanoparticles with a solid particle matrix are derived from O/W (oil/water) emulsions by the replacement of the liquid lipid (oil) by a solid lipid. These lipids are usually physiological lipids (biocompatible) with low toxicity [3]. SLNs are composed of lipids which are solid at room and body temperatures. The main advantages of SLNs are their high encapsulation efficiency, possibility in large-scale production, their flexibility in the controlled release profile due to the solid matrix, and their high ability to reach the target organ. However, SLNs can crystallize, allowing a very small space for oil incorporation and, thus, a low loading capacity [31]. The lipid nanoparticle diameters can be in the range between 50 nm and 1 μm [3]. SLNs have a low encapsulation load and a possibility of explosion during storage [31].

Rice bran oil nanocapsules were synthetized using poly(ε-caprolactone) (PCL) as wall material to evaluate their protective effect against UVB radiation-induced skin injury in mice, and the authors concluded that rice bran nanocapsules (200 nm, potential zeta of − 9 mV and a low polydispersity index (PDI) of < 0.2) inhibited 60% of the edema induced by UVB irradiation [32].

Oehlke et al. [33] prepared SLNs with ferulic acid (FA) and tocopherol (Toc). The different formulations, containing up to 2.8 mg g^−1^ of FA or Toc, were stable for at least 15 weeks of storage at room temperature. The authors concluded that these SLNs are suitable as food additives where a gradual release of the active compound could be beneficial.

### Trends in Oil Nanoencapsulation

**Fig. 1:**
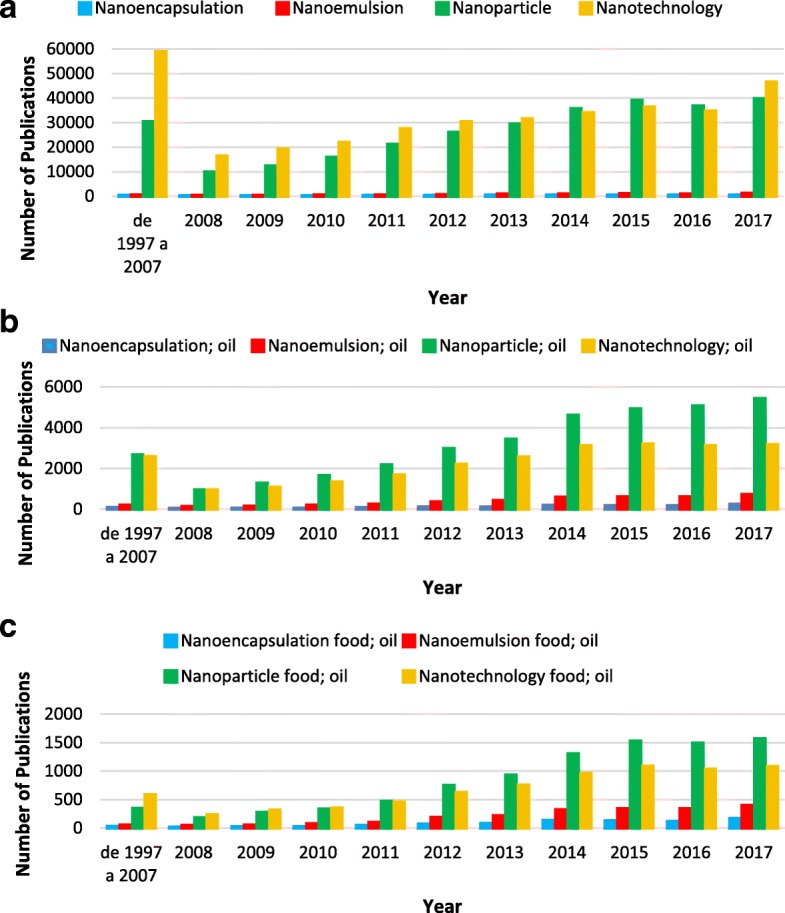
Number of nanoencapsulation, nanoemulsion, nanoparticle, and nanotechnology publications per year in the Scopus database using the following keywords: **a** nanoencapsulation, nanoemulsion, nanoparticle, and nanotechnology; **b** nanoencapsulation and oil, nanoemulsion and oil, nanoparticle and oil, and nanotechnology and oil; and **c** nanoencapsulation and food and oil, nanoemulsion and food and oil, nanoparticle and food and oil, and nanotechnology and food and oil

Many publications from the last 20 years contain the 4 terms nanoencapsulation, nanoemulsion, nanoparticle, and nanotechnology (Fig. 1). However, before the 2000s, articles containing these four terms regarding the research in oil and food applications initiated in the late 1990s constituted less than 2% of the examined publications, making this topic a small sector of nanotechnology (Fig. 1).

**Fig. 2:**
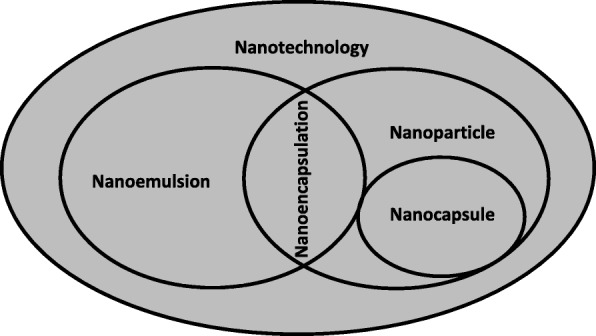
Scheme of the nanoencapsulation definitions commonly used for oils

The term nanotechnology was used in many publications as a more general term (Fig. 2). When using the combination of these terms and “oil” (Fig. 1b), an increase in publications involving the term “nanoparticles” is observed. The number of publications involving “nanoemulsion” and “oils” have increased significantly since 2010, either in general areas or those related to food (Fig. 1b).

Although there are far more publications involving “nanoparticles” and “nanotechnology” (Fig. 1a), encapsulation is the most appropriate term used to describe the packaging of substances into micro- and nanoparticles and is defined as a process involving one substance, referred to as the “active agent,” within another product referred to as the “wall material” [34–36].

Most publications on oil nanoencapsulation use the term “nanoencapsulation” [2, 37–42] or “nanoemulsion” [10, 43–48]. Some authors use the term “nanocapsules” [49–51], and others use “nanoparticles” [35, 41]. However, both terms originally mean “nanoencapsulation” (Fig. 2), which has been used in its broadest sense, encompassing both the nanocapsules and nanoparticles formation [52].

The term “nanoparticle” is a collective name for both nanospheres and nanocapsules [17]. Nanocapsules possess a polymeric membrane with a liquid nucleus, in which the active compound is confined to a cavity consisting of an inner liquid core surrounded by a polymeric membrane (the core shell structure can be lipophilic or hydrophilic) [3, 17]. On the other hand, nanospheres can be defined as solid colloidal fragments in which bioactive composts are diffuse, captured, encapsulated, and chemically chained to or adsorbed into the polymer matrix. The polymer matrix forms a porous or solid matrix, and the core can likely turn into a solid material relying on the copolymer structure [3, 53]. Nanoparticles are usually coated with nonionic surfactants to reduce immunological interactions and help reduce molecular interactions of the chemical groups on the particle surface (van der Waals, hydrogen bonding, or hydrophobic interactions). The intracellular uptake of nanoparticles is higher than that of other encapsulated systems. According to the applied methodology, nanocapsules may act as a vehicle to the retained active material to the polymeric inner membrane. The oil released from these systems can be transported from the nanoparticles to the target tissue by desorption, diffusion, or erosion [3].

Nanoemulsion is the beginning of nanoencapsulation, a system formed by oily and aqueous phases and the emulsification of these phases through the use of an emulsifier. In addition, nanoemulsions are formed with small drop sizes and high surface areas [10, 18, 37, 54]. Such properties grant them potential advantages over conventional emulsions, such as a good physical stability and higher bioavailability [10, 19].

The first nanotechnology definition was discussed in 1959 by the renowned physicist Richard Feynman in his talk *There is Plenty of Room at the Bottom*, in which he described the possibility of synthesis via direct atom manipulation. “Nanotechnology” was first used by Norio Taniguchi in 1974. Nanotechnology emerged as a field in the 1980s, and from this time forward, there has been an increase in scientific publications and awareness in the area; research in this area intensified in the 2000s (Fig. 1), as did scientific, political, and commercial attention, leading to both controversy and progress. Furthermore, product commercialization based on the advancements in nanoscale technologies began to emerge [55].

Nanotechnology is a multidisciplinary field that covers a vast range of materials, processes, and applications and encompasses chemical, physical, biological, electronic, and engineering sciences. It focuses on the fabrication, characterization, and experiment of substances at the nanoscale range, almost between 1 and 100 nm. The minimum particle size, in relation to the growth surface area, exhibits unique and novel properties and creates vast potential for technological uses [55–57].

Nanotechnology can advance strategies for thermal and storage stability, water solubility and bioactive substances, increase the bioavailability for food usage, and enhance the macroscale properties of foods, such as taste, texture, industrial processes, and coloring strength [58]. The major food companies have used their own research departments to design strategies for applying nanotechnologies in functional foods [59].

### Current State of Oil Nanoencapsulation Applications

The growth of the food discipline is quantified in Fig. 1b, c as the aggregate number of publications containing the keywords “food” and “oil” and “nanoencapsulation,” “nanoemulsion,” “nanoparticle,” or “nanotechnology” in their abstract; the information is shown as a function of the publishing year. As indicated by the trends in Fig. 1, most of the growth in the food nanotechnology field took place after the year 2010 because of the numerous nanotechnology studies of the late nineties and the growth of food-grade additives suitable for the nanoparticle process. Industry oil nanoencapsulation applications are summarized in Table 1.

**Table 1:** Summary of some oil nanoencapsulation applications in the food industry

Principle “nano” component	Function	Future applications	References
Normal oils			
Fish oil	Yogurt, fruit juice, and other food enrichment; protection against oxidation and strong odors	Foods with superior nutritional value and with high bioavailability; product approach containing no saturated fatty acids but rich in unsaturated fatty acids; beverage industry or as a food seasoning, for example, in fish sauce	[34, 35, 41, 42, 44, 86]
High-oleic palm oil (HOPO)	Obtain the most favorable microfluidization, formation and storage conditions for the nanoemulsions obtained from HOPO	Potentiate its use in the food industry to preserve the oils’ favorable properties	[10]
Chia seed oil	Protect the oil against lipid oxidation and improve the solubility and stability	Chia mucilage can be used as a wall material in bioactive oil nanoencapsulations for application in the food area to substitute synthetic polymers	[37]
Orange oil	Fabricating flavor oil delivery systems using rapid and simple processing operations.	Utilization in foods and beverages	[43]
Roasted coffee oil	Stabilize the roasted coffee oil flavoring compounds, or even to promote their controlled release	Improve the coffee aroma	[83, 84]
Essential oils			
Thyme essential oil	Control the lipid oxidation, microbial spoilage, and sensory change of fresh beef burgers during chilled storage	Improve the microbial, chemical, and sensory quality of meets, for example, and enhance food safety	[28, 87]
*Eucalyptus staigeriana* essential oil	Antimicrobial activity against *Salmonella enteritidis* and *Listeria monocytogenes*	Good alternative for natural food preservation	[82]
Oregano essential oil	Antimicrobial activity in chicken pâté	Food formulations, replacing harmful synthetic food preservatives	[80]
A terpenes mixture extracted from *Melaleuca alternifolia* and d-limonene essential oil	Enhance the antimicrobial activity in fruit juices	Improve the safety and quality of foods through the addition of natural preservatives	[81]
Cinnamon essential oil	Retard beef patty deterioration during refrigerated storage	Looks promising to lower lipid oxidation and microbial growth and increase red color stability in beef patties. Can provide support to expand the use of nanoencapsulation techniques in the design of delivery systems for the utilization of natural preservatives in functional food	[79]

Currently, nanotechnology products in the food industry reach a value of US$1 billion (mostly consisting of nanoparticle coatings for health promoting products, packaging technologies, and drafts), and they have the chance to increase more than US$20 billion in the next 10 years. Many reviews show an excellent summary of the research groups and private and public organizations that have been leading the food nanotechnology field [11, 13, 60].

Although a number of reviews [11, 13, 55, 60, 61] have discussed the food nanotechnology investment and the emerging applications of nanotechnology for primary production, there are no reviews addressing oil nanoencapsulation when considering oil as the encapsulated material. Furthermore, there are many reviews on nanotechnology applications [13, 53, 55, 61–68], and most of them focus on nanotechnology in food applications [13, 52, 55, 61–68].

In the food industry, the microencapsulation process can be used for a variety of reasons, which have been summarized by Desai and Park [4] as follows: (a) the core material is protected from degradation by reducing its responses to the external environment; (b) the evaporation or transfer rate of the main material to the external environment is decreased; (c) the physical characteristics of the original material is modified to allow easier handling; (d) the release of the core material is tailored to occur slowly over time or at a particular time; (e) the unwanted flavors or tastes of the core material are masked; (f) equal dispersion in the keeper material is achieved; and (g) the mixture components that would otherwise react with one another are separated. These applications are also suitable for oil nanoencapsulation. Ricaurte et al. [10] and Campo et al. [37] studied high-oleic palm oil (HOPO) and chia seed oil with different aims. The first study aimed to find the most favorable microfluidization, formation, and storage conditions for the nanoemulsions obtained from HOPO and the second study promised alternatives to protect the oil against lipid oxidation and improve solubility and stability (Table 1).

Cushen et al. [9] affirms that the above assertion that food microencapsulation is well established; microencapsulated fish oil has been applied in bread for functional healthy benefits. The microencapsulating process masks the unpleasant taste of fish oil, and this bread is already feasible in the market. The nanoencapsulation and addition of compounds in the food industry is a logical progression of the technology [2, 68]. Furthermore, oxidation reactions, the main deterioration processes of fats, oils, and lipid-based foods, result in decreased nutritional value and sensory quality, and oil nanoencapsulation promotes the reduction in oxidation through the formation of protective barriers formed during the nanoencapsulation process, as previously stated [2].

In their review, Walker, et al. [47] highlighted the promise of using nanoemulsions for the encapsulation, security, and release of omega-3 fatty acids. These carry systems can be used in the food industry in beverages with these bioactive lipids and to fortify foods, or they may be used in the supplement or pharmaceutical industry to enhance the bioactivity of functional omega-3 fatty acid compositions.

Sozer and Kokini [67] simplified nanotechnology use in the food and food-packing industries. Types of food benefits included protection against oxidation; controlled release of encapsulated ingredients (moisture or pH); test disguising; delivery of nanoencapsulated nutrient substances, vitamins, and flavors; pathogen detection in food systems; food safety; and quality analysis. Some food packaging applications included improved packaging (gas and moisture barriers, tensile strength); shelf-life extension via active packaging, nanoadditives, intelligent packaging, nutraceutical delivery, and controlled release; antibacterial effects of self-cleaning packaging; and product condition monitoring during transportation. Applications in food packaging are considered highly promising because they can enhance the safety and quality of the food products. These applications include intelligent packaging, which is able to interact with the food product. However, for oil nanoencapsulation application in the food industry, fish oil is normally used, and the purpose of the nanoencapsulation is to primarily protect the oil from lipid oxidation for food fortification [34, 38, 40].

As can be seen, fish oil is the most used oil in both micro and nanoencapsulation. It is a source of unsaturated and PUFA. Humans can produce the majority of fatty acids. Nevertheless, omega-6 (n-6) and omega-3 (n-3) fatty acids, which are essential in human nutrition, cannot be synthetized by the human organism. Thus, humans have to acquire them from food. The intake of vegetable oils (edible oils), including PUFA, is related to a low incidence of chronicle diseases, such as cardiovascular or neurological disorders, and a decrease in cancer rates [3, 69].

Bioactive oils are usually applied for their nutritional properties, but one of the main problems in relation to their use is the loss of active components during storage [70]. This occurs because bioactive oils contain PUFA and other substances (xanthophylls, sterols, carotenoids, monoterpenes, flavonols, etc.) sensitive to oxygen, moisture, heat, and light [71]. The products formed in oxidized oils include numerous free radical species, primary oxidation products such as lipid hydroperoxides, and secondary oxidation products such as hydrocarbons, aldehydes, epoxides, and ketones. Some of these products can negatively affect biological tissues [72]. Because of this oxidation, the properties and nutritional value of the oil are lost and an unpleasant taste and odor result [3].

The other active compounds in these oils can exhibit antioxidant, anti-inflammatory, antiviral, antibacterial, anticancer, and/or tissue regenerative properties [73]. The polyphenols and tocopherols in oils exhibit an important antioxidant activity. Hence, the characteristics and composition of antioxidants vary according to oil type. Accordingly, olive, sunflower, argan, and grape seed oils contain high contents of antioxidant compounds [72]. In addition, the presence of labile compounds such as sterols, carotenoids, xanthophyll, flavonols, and monoterpenes also contributes to the nutritional value and health properties of an oil [3].

Moreover, EOs are common plant products composed of mixtures of biologically active materials, and they provide potentially bioactive compounds and novel molecule templates [74, 75]. EOs are composed of volatile secondary metabolites with antifungal, antibacterial, antioxidant, anti-inflammatory, antiviral, and anticancer activities [76]. EO efficiency depends on its chemical composition, genotype, and environmental and agronomic conditions [77]. Some examples of these oils are thyme, lavender, peppermint, cinnamon, tea tree, rosemary, eucalyptus, and lemongrass oil, as well as a few others. These oils have been shown to exhibit antimicrobial properties but are extremely vulnerable to oxidation [15, 27, 78].

EOs are classified as natural bioactive molecules deemed suitable for use in the inhibition of the growth of foodborne pathogens. However, the direct incorporation of EOs into food presents technological challenges due to the high volatility of some EO constituents, the difficulty of EO incorporation into aqueous formulations, and the possibility of drastic changes in the sensory properties of food products. Among the components that exhibit antimicrobial activity, oregano, carvacrol, thymol, and γ-terpinene have been used in food.

Some essential oils have been used to improve the microbial, sensory, and chemical quality of foods such as meat, chicken, and fruit juices [28, 79–81]. Ghaderi-Ghahfarokhi et al. [28] nanoencapsulated thyme essential oil and used it in beef burger. They observed that the encapsulation process improved the shelf-life of the thyme essential oil and minimized the vaporization of active compounds at the beginning of storage. In addition, the slow release of the thyme essential oil during storage could maintain, or even increase, the antioxidant and antimicrobial activity of the oil until the end of refrigerated storage. In addition, there were positive changes in the redness and oxymyoglobin content from burger compared to that of the controls, and free thyme essential oil improved the acceptability and sensory quality of beef burgers.

There are studies that used essential oils in food as natural preservatives to improve food safety and quality, replacing harmful synthetic food preservatives [49, 82]. Herculano et al. [82] encapsulated eucalyptus and determined the antimicrobial actions of the loaded nanoparticles on *Listeria monocytogenes* and *Salmonella enteritidis* bacteria. The authors observed that the nanoparticle bactericidal action was more effective against gram-positive than gram-negative bacteria, as the nanoencapsulated oil exhibited enhanced activity against *S*. *enteritidis*; these nanoparticles can be used in foods for natural preservation.

Cashew gum (CG), whose structure resembles Arabic gum, is a heteropolysaccharide extracted from the exudate of *Anacardium occidentale*, a tree common in the Brazilian northeast region. Cashew gum is able to interact with water and thus act as a stabilizer, emulsifier, and adhesive and could be a good substitute for gum arabic, which is more expensive. CG was used by Herculano et al. [82] to encapsulate *Eucalyptus staigeriana* essential oil (ESO), and the diameter (nm) and zeta potential (mV) of the capsules from the formulation were, respectively, F1: 153.80 ± 8.20 and − 24.50 ± 0.45; F2: 27.70 ± 3.42; − 14.47 ± 1.42, and F3: 432.67 ± 41.47; − 10.45 ± 0.21. These formulations were composed of F1: CG: ESO = 2:1; ESO: Tween 80 = 2:1; F2: CG: ESO = 4:1; ESO: Tween 80 = 2:1; F3: CG: ESO = 2:1; ESO: Tween 80 = 1:1. The F1 and F2 samples showed a unimodal distribution, whereas F3 had a bimodal distribution (nano- and microparticles).

### Nanoencapsulation Methods Applied in Different Oils

**Fig. 3:**
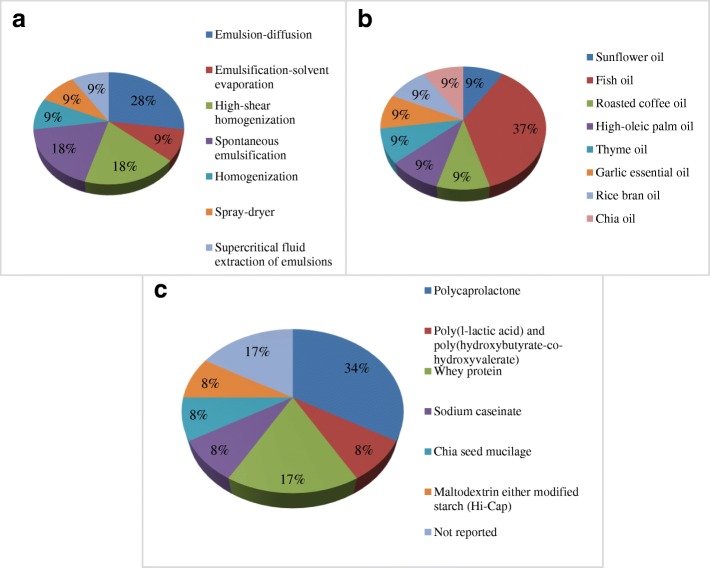
Proposal and techniques (**a**), employed oils (**b**), and wall materials (**c**) of some studies using oil nanoencapsulation in food

In this review, 11 studies that used nanoencapsulated oils in the food industry were analyzed [10, 16, 35, 37, 38, 83–87], and 1 figure, Fig. 3 was made that describes the technologies, nanoencapsulated oils, and wall materials used. Generally, there are many methodologies for the production of nanocapsules containing oils, such as emulsion-diffusion [16, 38, 85], emulsification-solvent evaporation [83], high-shear emulsification [10, 87], spontaneous emulsification [84, 88], homogenization [37], spray drying [35], and the emulsion supercritical fluid extraction process [86] (Fig. 3a). In general, the techniques are similar, with some particular similarities between each of them.

In emulsion-diffusion, an emulsion is produced after a dilution causes the deposition of a polymer around the droplets, whereas in emulsification-solvent evaporation, an emulsion is formed with a polymer solution and an aqueous phase. The solvent is evaporated at the end of both techniques. High-shear homogenization, or microfluidization, is a kind of high-energy emulsification which uses microfluidizers to create mechanical shear. This equipment works by dividing a liquid jet into two parts. Every part passes through a narrow opening. Normally, emulsions with a diameter greater than 1 μm are first formed by other methods, after which their sizes are then reduced in a microfluidizer [3].

Spontaneous emulsification, or low-energy emulsification or self-emulsification, is a process which depends on different variables: interfacial and bulk viscosity, interfacial tension, phase transition region, and surfactant structure and concentration because the emulsion is formed spontaneously as a result of the low interfacial tension from high surfactant levels. In the homogenization (nonspecific name) technique, the emulsion is composed of an organic phase, which has a surfactant, organic solvent and oil, and of an aqueous phase, which is composed of water and a polymer. The organic phase is added dropwise to an aqueous solution. Then, the solvent is removed by a vacuum process [37].

The spray dryer technique is based on dissolving or dispersing the active ingredient in a biopolymer solution. Then, the dispersion is atomized in a heated air chamber that rapidly removes the solvent and produces a dried particle consisting of the active ingredient embedded in a porous wall material [31]. The supercritical fluid extraction of emulsions (SFEE) technique is based on the use of supercritical carbon dioxide (CO_2_) to rapidly extract the organic solvent from an oil-in-water emulsion, in which a bioactive compound and its coating polymer have been previously dissolved. By removing the solvent, both compounds precipitate, generating a suspension of particles in water [86].

In addition to that in oil microencapsulation, the oil usually used in nanoencapsulation is fish oil [16, 35, 38, 86] (Fig. 3b). However, sunflower oil [85], roasted coffee oil [83], HOPO [10], thyme oil [87], garlic essential oil [84], rice bran oil [88], and chia oil [37] are also used (Fig. 3b).

Oil nanocapsules have been produced with the application of different wall materials (Fig. 3c), depending on the usage and kind of oil to be nanoencapsulated. Unlike oil microencapsulation, oil nanoencapsulation does not usually involve wall material mixtures. Usually, the wall material is used alone because the particles formed must have a size of 1000 nm, and, as there is a larger contact surface, the fewer the compounds in the nanocapsule formulation the better the interaction is among the compounds, ultimately favoring the particle size.

The wall materials most used in the techniques are biodegradable polymers. Some usual wall materials used in oil nanoencapsulation are polycaprolactone [16, 38, 86, 88], whey protein [10, 35], sodium caseinate [87], chia seed mucilage [37], maltodextrin, or modified starch [35] (Fig. 3c). Some authors did not report the wall material used in their study [88], probably because of the spontaneous emulsification technique that was employed.

Wall material is chosen according to the size of the required nanoparticles, aqueous solubility and stability, and other factors. Among polymers, most of the wall material utilized is poly(ε-caprolactone) (PCL). PCL is a polymer obtained through the ring-opening polymerization of the cyclic monomer Ɛ-caprolactone utilizing cationic or anionic, coordination, or the radical catalysts mechanism. This polymer is semicrystalline, and its crystallinity is directly associated with its molecular weight. It is soluble in inorganic solvents and has a good blend compatibility that provides a transformation of chemical properties, such as solubility and porosity, and it presents a low melting point (59–64 °C). Furthermore, PCL is a synthetic, biocompatible, and fully biodegradable polymer that has a semi crystalline nature (glass transition temperature of 213 K). It is approved for drug delivery by the Food and Drug Administration (FDA). Due to its slow degradation, PCL is ideally suited for long-term delivery or when a targeted delivery to the intestinal tract is intended. PCL has a high hydrophobicity, high in vitro stability, and low cost [87]. Usually, PCL is utilized in the emulsion-diffusion method and supercritical fluid extraction of emulsions, especially for fish oil encapsulation [16, 86].

Whey protein may also be applied to nanoencapsulate bioactive compounds such as oils because of its functional characteristics, such as its surface activity, gelation, shielding, and protective properties, e.g., biocompatibility and biodegradability [58]. Ricaurte et al. [10] applied HOPO and obtained nanocapsules with whey protein from microfluidization, confirming that this methodology was able to create stable nanocapsules with a diameter of 163 nm.

After synthesis, the basic characterization of the oil nanoparticles is determined by important parameters, such as the size, polydispersity index (PDI), and zeta potential. The size and size dispersion of nanocapsules are important because of their ability to transform the physicochemical and pharmaceutical behaviors of the encapsulated ingredients [58].

Nanoparticle size, also named the mean diameter or z-average, may be established by several methods, such via laser diffraction (LD) and a Coulter counter; however, the most applied technique is dynamic light scattering (DLS) [58, 89], which allows the description of particle size distribution and destabilization phenomena. Nevertheless, it is not very precise when used with large size differences; it is noted that particles larger than 1 μm will be subject to gravitational movement in addition to Brownian motion, which makes this technique suitable for the characterization of particles only < 1 μm.

For nanoencapsulated oils, the diameter size is usually between 100 and 1000 nm [10, 16, 35, 37, 38, 83, 85, 87] or less than 100 nm [84, 86–88]. Diameters larger than 1000 nm were found by Ricaurte et al. [10]; those authors reported diameters between 163 and 2268 nm using the microfluidization method and whey protein as a wall material in the nanoencapsulation of HOPO.

Size dispersion is indicated as the PDI, an index that describes the particles uniformity in suspension; PDI values between 0.1 and 0.25 [10, 38, 87, 88] indicate a small size distribution, and PDI values higher than 0.5 indicate a broad distribution [50]. Although some authors, such as Choi et al. [16], Campo et al. [37], and Jafari et al. [35], did not report PDI, it is a good parameter for characterizing nanoparticles when used with particle size and zeta potential. Campo et al. [37] did not perform PDI analysis, but they found a bimodal figure in one of the diameter size results, suggesting the presence of nano and microparticles; if PDI was performed, the values would likely be greater than 0.25.

Zeta potential is a physical characteristic that is shown by particles in suspension, macromolecules, or substance surfaces; it corresponds to the nanoparticle’s electrical potential, as influenced by the nanocapsule ingredients and the medium in which they are distributed. This parameter is widely applied to indicate suspension stability in colloidal dispersions, where zeta potential values higher than 30 mV and lower than − 30 mV promote high stability and prevent particles aggregation [90]. The majority of the studies examined here obtained results between these values (30 mV and − 30 mV) [10, 37, 38, 84, 85]. Some authors, such as Choi et al. [16], Freiberger et al. [83], Bernardi et al. [88], Jafari et al. [35], and Pietro and Calvo [86], did not report the zeta potential.

For nanoencapsulated oils, the zeta potential is usually variable because of wall material characteristics. Campo et al. [37] obtained a zeta potential of − 11.58 ± 1.87 mV for encapsulated chia oil with chia seed mucilage as wall material. Nanoparticles of anionic gums, such as chia seed polysaccharide and cashew gum, can present negative zeta potential due to the presence of carboxylic acids groups in the carboxylate form (-COO-) that generates negative charges [82].

Another important analysis for the characterization of nanoparticles is Fourier-transform infrared spectroscopy (FTIR), which is a technique used to obtain an infrared spectrum of the absorption or emission of a solid, liquid or gas. An FTIR spectrometer simultaneously collects high spectral resolution data over a wide spectral range. This provides a significant advantage over a dispersive spectrometer, which measures intensity over a narrow wavelength range. FTIR is a less intuitive way to obtain the same information. Usually, oil nanoparticles are used in the transmittance mode, operating with wavelengths between 400 and 500 and 4000 cm^−1^ and a resolution of 4 cm^−1^ [37, 84, 91].

Based on FTIR analysis, it is possible to physically perceive the interactions that take place between the nanoparticle components; for example, the FTIR results of nanoencapsulated garlic essential oil showed the characteristic Tween 80 (the emulsifier used) peaks. This phenomenon could be related to coverage in the garlic oil nanoemulsion spectrum due to the stretching vibration of the extracted garlic bands. The band at 1325–1450 cm^−1^ showed the presence of S=O, and the band at 1675–1600 cm^−1^ showed a -C-C=C symmetric stretch, both of which are present in garlic EO compounds [84].

### Incorporation of Nanoencapsulated Oils into the Food Market and Patent Application

According to the House of Lords [92], food currently contains structures at the micro and nanoscale. Fruit juice is composed by plant material that was built from nanoscale ingredients, while Bailey’s Irish Cream contained nanoemulsions with an average droplet size of 190 nm. Margarine had water droplets smaller than 10 μm across, with even smaller fat crystals interspersed in them. The naturally occurring nanomaterials found in food ranged from particles smaller than 100 nm found in drinks such as tea, beer, and coffee to protein structures of approximately 300 nm found in eggs or soy to larger oil particles of approximately 800 nm found in substances such as milk. All fresh and processed food was structured at the nanoscale, and consequently, the body evolved over time to deal with nanoscaled materials.

Few studies have been performed on the incorporation of nanotechnology incorporation into trade [65]. Furthermore, no products that contained oil nanoencapsulation were found in the market. However, there are numerous oil microencapsulation products that can be found in trade, and there is an article that highlights this information [93]. This finding may be attributed to the fact that, in general, nanotechnology is relatively new, and it is a relatively complex technology to employ. However, it is possible to notice some similarities between the methods used for the micro and nanoencapsulation of oils. In addition, the regulation gap in nanotechnology raises some uncertainties about the use of this technology in the market.

Concerning nanotechnology regulation, there are a number of ongoing EU research projects aimed at addressing all aspects of nanosafety, including toxicology, ecotoxicology, risk assessment, exposure assessment, mechanisms of interaction, and standardization. Examples of ongoing EU projects include the NanoLyse project, which is dedicated to the development of analytical tools for the detection and characterization of engineered nanoparticles in food, and the NanoReTox project, which seeks to address the human health and environmental implications of exposure to engineered nanoparticles [94]. However, regulatory institutions such as the Environmental Protection Agency (EPA) and the Food and Drug Administration (FDA) in the USA or the Health and Consumer Protection Directorate of the European Commission have started addressing the potential risks posed by nanoparticles. So far, neither engineered nanoparticles nor the products and materials that contain them are subject to any special regulation regarding production, handling, or labeling.

Although there is no specific nanoparticle regulation, there are some food industry patent documents deposited in different countries. WO2018029626, a patent application from Argentina, focused on chia oil with an edible nanoemulsion. It described a chia oil nanoemulsion comprising between 10 and 20% of chia oil (*Salvia hispanica L.*), between 2 and 5% of polysorbate, between 0.5 and 5% of at least one emulsifier other than the polysorbate, between 0.05 and 0.2% of at least one antioxidant, and water. Formulations of edible chia oil nanoemulsions used in transparent drinks and desserts, such as juices and jellies, were disclosed [95]. A patent application from the Republic of Korea, KR20160005182, focused on cinnamon oil nanoemulsions to inhibit the development and increase of dangerous food microorganisms. Furthermore, this invention could not only be used for food additives, food packaging materials, preservatives, etc. but also be utilized in the pharmaceutical and cosmetic industries [96]. A mustard oil nanoemulsion application patent from China, CN103315956, was prepared to alleviate the pungent smell of mustard oil to avoid volatilization, and the mustard oil may be used for bacterial resistance in food and drugs [97]. Wang Weichun Feng Wei submitted an application patent from China, CN103750050, describing a palm oil nanoemulsion that solved the problems of high grease costs, low absorption rates, low oil content in the existing prepared palm oil nanoemulsions, large granularity, poor stability, long production periods, high equipment investments, and high production costs in existing young animal feeds. The palm oil nanoemulsion was prepared by mixing an emulsifier with palm oil, cutting and emulsifying the mixture, and ultrasonically performing cell breaking in the mixture. The process was simple, the entire reaction process was easily controlled, the entire process production period was short, the equipment investment and production costs were low, the oil content of the produced nanoemulsion was high, the distribution granularity was small, the stability was good, and the digestion by livestock increased [98].

There is a growing trend of oil nanoencapsulation patent applications, indicating that many innovations have been made and attesting to the technology the global market.

## Conclusion

Nanoencapsulation is well-established for oil preservation. It offers a plethora of advantages, including the effective protection of the encapsulated oil against degradation, the possibility of accurate control of the oil release, easy administration, and avoidance of the evaporation of the volatile components. Moreover, nanoencapsulation may be achieved by a variety of techniques. Technique selection will depend on the physicochemical characteristics of the active compounds, the processing conditions, particle size and density necessary to incorporate the oil properly into the final product, the mechanism of release, and the cost constraints. Although there currently are not many oil nanoencapsulation products in the food market, there is no doubt that if boosted by recent remarkable scientific advances, new approaches in oil nanoencapsulation will soon be considered in the application of oils in food additives and nutritional supplements, and patents application will continue to increase.

## Abbreviations

CG: Cashew gum
CO_2_: Carbon dioxide
DHA: Docosahexaenoic acid
DLS: Dynamic light scattering
EOs: Essential oils
EPA: Eicosapentaenoic acid
ESO: *Eucalyptus staigeriana* essential oil
FA: Ferulic acid
FDA: Food and Drug Administration
FTIR: Fourier-transform infrared spectroscopy
HOPO: High-oleic palm oil
LD: Laser diffraction
n-3: Omega-3 fatty acids
n-6: Omega-6 fatty acids
PCL: Poly(ε-caprolactone)
PDI: Polydispersity index
PUFA: Polyunsaturated fatty acids
SFEE: Supercritical fluid extraction of emulsions
SLN: Solid lipid nanoparticles
Toc: Tocopherol
US EPA: United States Environmental Protection Agency
UV: Ultraviolet radiation
